# Development and Evaluation of a Web-Based Paediatric Drug Information System for Germany

**DOI:** 10.3390/pharmacy9010008

**Published:** 2021-01-05

**Authors:** Julia Zahn, Stefan Wimmer, Wolfgang Rödle, Irmgard Toni, Brita Sedlmayr, Hans-Ulrich Prokosch, Wolfgang Rascher, Antje Neubert

**Affiliations:** 1 Department of Paediatrics and Adolescent Medicine, Faculty of Medicine, Universitätsklinikum Erlangen, Friedrich-Alexander-Universität Erlangen-Nürnberg (FAU), 91054 Erlangen, Germany; stefan.wimmer@uk-erlangen.de (S.W.); irmgard.toni@uk-erlangen.de (I.T.); Wolfgang.Rascher@extern.uk-erlangen.de (W.R.); 2 Department of Pharmacy, Universitätsklinikum Erlangen, Friedrich-Alexander-Universität Erlangen-Nürnberg (FAU), 91054 Erlangen, Germany; 3 Chair of Medical Informatics, Friedrich-Alexander-Universität Erlangen-Nürnberg (FAU), 91058 Erlangen, Germany; wolfgang.roedle@fau.de (W.R.); Brita.Sedlmayr@uniklinikum-dresden.de (B.S.); hans-ulrich.prokosch@fau.de (H.-U.P.); 4 Institute for Medical Informatics and Biometry, Carl Gustav Carus Faculty of Medicine, Technische Universität Dresden, 01069 Dresden, Germany

**Keywords:** evidence-based medicine, off-label use, drug formulary, paediatric pharmacotherapy, paediatrics, medication safety, information technology

## Abstract

**Background:** Off-label use is frequent in paediatrics but that does not necessarily mean that the risk-benefit ratio is negative. Nevertheless, evidence-based data is essential for safe drug therapy. In Germany, there is no publicly available compendium providing transparent, evidence-based information for paediatric pharmacotherapy to date. This work describes the development of a web-based paediatric drug information system (PDIS) for Germany and its evaluation by health care professionals (HCP). **Methods:** Since 2012, a PDIS is being developed by the authors and is supported by the Federal Ministry of Health since 2016. Dosing recommendations were established based on systematic literature reviews and subsequent evaluation by clinical experts. The prototype was evaluated by HCP. Based on the results, the further development was concluded. **Results:** 92% of HCP believed that the PDIS could improve the quality of prescribing, as currently available information is deficient. Besides the license and formulations, dosing recommendations were the most relevant modules. A dosage calculator was the most wanted improvement. To facilitate sustainability of future development, a collaboration with the Dutch Kinderformularium was established. As of 2021, the database will be available to German HCP. **Conclusion:** The fundamentals for a German PDIS were established, and vital steps were taken towards successful continuation.

## 1. Introduction

Due to the lack of evidence from valuable clinical studies, and because drug development historically focused on adults, children have reduced access to licensed medicines leading to high off-label (up to 79%) and unlicensed use (up to 15%) [1]. Children are, therefore, at increased risk for adverse drug reactions and medication errors [2]. The possibility of experiencing harm from an adverse effect is three times greater in the paediatric population compared to adults [3,4]. Most of the severe medication errors are caused by errors in drug administration (41%) and prescribing (32%); thus, being potentially preventable [3].

Regulatory efforts improving the development and accessibility of appropriate medicines for children have been initiated worldwide within the past decades. However, the expected improvement for generic drugs frequently used off-label in children has not been realised. Only six off-patent medicines were licensed via the Paediatric Use Marketing Authorisation (PUMA) route within more than ten years so far [5].

The absence of a paediatric license does not necessarily mean that the risk-benefit ratio of a drug in a paediatric age group is negative. For some drugs and indications, data from, e.g., clinical trials are available, and a well-established, evidence-based use can be assumed. On the other hand, a paediatric license does not imply high-level evidence for the use in children and that the latest scientific data are integrated into the license [6,7]. Furthermore, off-label use leads to significant variability of therapeutic approaches [8,9]. For example, more than ten different dosing regimens are recommended in Europe for ciprofloxacin and fluconazole [10].

Paediatric drug formularies are an essential source of information regarding the use of medicines for children [7]. They are needed to support the rational use of drugs, particularly when they are used outside the terms of their license, and to move away from practice-based, empirical to systematic and evidence-based pharmacotherapy [11]. A few well-established evidence-based paediatric formularies are available across Europe, such as the British National Formulary for Children (BNFc), the Dutch Paediatric Formulary (Kinderformularium.nl) or the Pediatric & Neonatal Dosage Handbook from the U.S. [12,13]. The two handbooks in English, the BNFc and the Pediatric & Neonatal Dosage Handbook are subject to a fee. Kinderformularium.nl is freely available but in Dutch language only. In Germany, however, there is no publicly available compendium providing transparent and evidence-based information for on- and off-label paediatric pharmacotherapy to date. Most of the existing pocketbooks consist of empirically collected information without indicating the source of information or the level of evidence. These details are, however, crucial to decide on rational drug therapy and at the same time to minimise the risk of drug therapy, especially for drugs being used off-label. To fill this gap, we developed a web-based platform containing evidence-based dosing recommendations as well as pharmacological and pharmaceutical information for drugs used in children in Germany.

The aim of this work is to describe the development and history of the paediatric drug information system (PDIS) for Germany and its evaluation by German healthcare professionals (HCP).

## 2. Methods

### 2.1. First Stage of the Development of the PDIS

Since 2012, the Department of Paediatrics and Adolescent Medicine of the University Hospital of Erlangen, Germany, has been establishing a database providing dosing recommendations for paediatric drug therapy. In 2016, the German Federal Ministry of Health funded the “PaedDos” project within the scope of the “Action Plan 2013–2015” to improve the safety of drug therapy in Germany. PaedDos aimed at the development and set-up of an evidence-based online formulary for paediatric drugs in Germany.

Standard operating procedures (SOP) for the research of drug information were issued to ensure the quality of the content provided by the PDIS. The Federal Ministry of Health grant facilitated a high scientific standard because one of the funding requirements was that dosing recommendations for off-label use must be based on systematic literature searches, including primary literature. Therefore, available data from clinical studies and case series in children have to be considered, and benefits of the drug use need to be weighed against risks before the most appropriate dosing regimen is proposed. The first 100 drug monographs included in the PDIS were based on a list of the most frequently prescribed drugs for patients under 18 years of age, according to out- and inpatient prescription data in Germany.

### 2.2. Online User Test

To identify the demand for a PDIS for Germany and to adapt the database to end-users’ needs, an online user test was developed by the project team comprising of pharmacists, paediatricians, and computer scientists. A questionnaire consisting of 90 items was designed in collaboration with a usability expert. Thirty items focused on the pharmacological, pharmaceutical part of the PDIS and are subject of the present analysis. The technical development of the prototype database and corresponding parts of the online user test has already been published elsewhere [14].

Participating paediatricians and pharmacists were recruited via e-mail or telephone. None of the participants was part of the project team nor had economic interests. During the user test, participants had full access to the web-based PDIS. The user test started with two scenarios for each occupational group [14]. Each scenario illustrated a situation in which the HCP could make use of the PDIS in daily practice. The scenarios had the aim to demonstrate the functionality and information provided by the database. In the second part of the user test, participants received the questionnaire, which was provided online via the SoSci Survey^®^ tool (https://soscisurvey.de/). Participants gave informed consent to anonymous collection and data processing digitally prior to participating in the online user test. Demographic information including occupation, professional experience in years, and number of drug prescriptions or administration advices per day were collected.

### 2.3. Data Analysis

For numerical evaluation of the answers of participants, a five-point Likert scale ranging from “does not apply at all” (1) to “completely applies” (5) was utilised [14]. The closer the selected numerical scale was to five, the higher the respondents’ agreement with the question. In the case of a ‘negative answer’ (<3), the user could add a further explanation in an open-ended field. If the question related to frequency, the answer options ranged from “never” (1) to “always” (5). Frequencies, means, and standard deviations were calculated.

### 2.4. Second Stage of the Development of the PDIS

Due to the early involvement of HCP representatives, specific requirements of end-users could be integrated into the development process of the PDIS. By doing so, it became evident that the underlying data structure of the original database was not suitable for an extension and that the integration of a dosage calculator was challenging to realise. Therefore, an evaluation of existing paediatric drug formularies was performed with regard to possible collaboration.

Since 2018, the Federal Ministry of Health is supporting the establishment of the PDIS with a second grant, intending to complete the database and provide the contents free of charge to German HCP.

## 3. Results

### 3.1. First Stage of the Development of the PDIS

A well-structured, web-based prototype of a PDIS was developed by the project team aiming to support HCP as best as possible within the medication process. The heart of the database consists of structured drug monographs comprising of six information modules (Table 1).

Primary literature of published data regarding the use of an active substance in any paediatric subgroup is selected via structured PubMed searches. Besides, the Summary of Product Characteristics (SmPC), as well as national and international guidelines from specialist societies, are considered and incorporated into the recommendations. In a final step, the dosing recommendation is reviewed by clinical experts of the relevant division prior to publication in the PDIS.

At the time of the online user test, the database contained about 100 drug monographs, all including dosing recommendations, licensing status, and references. Fifty of those monographs also included information on the remaining modules (e.g., pharmaceutical formulations, adverse drug events, warnings). Out of them, about ten were used for the scenarios of the online user test.

### 3.2. Online User Test

For participation in the online user test, 24 HCP were contacted. Response rates ranged between 43% (physicians in private practice) and 75% (pharmacists). Twelve HCP took part: six paediatricians in private practice, three clinicians from children’s hospitals, and three pharmacists (hospital: *n* = 1, public: *n* = 2). Physicians in private practice had the highest professional experience and most prescriptions per day (Table 2).

None of the participants had already used similar paediatric-specific online systems before, e.g., Kinderformularium.nl or Zak-Kinderarzneimittel. Eleven participants were less satisfied with the currently available information regarding paediatric pharmacotherapy and highlighted that better information should be made available.

Ninety-two per cent of the participants believed that the web-based platform has the potential to improve the quality of prescribing medicines to children and adolescents. Seventy-five per cent thought that the number of adverse events could be reduced by using such an information system. All participants declared that they would most likely use the database if it became available.

While most participants agreed that the platform’s visual appearance is good, a minority mentioned that essential information should be highlighted by colour coding or using symbols, e.g., severe child-specific side effects. Four participants wished for better support in choosing the correct dose by providing dosing lists based on age/weight categories or a dosage calculator (Table 3). Eleven out of twelve participants answered that they would use an automatic dosage calculator if available on the platform. The remaining participant (physician in private practice) commented that an automatic dosage calculator conveys false safety wherefore he would rather not use it.

**Figure 1:**
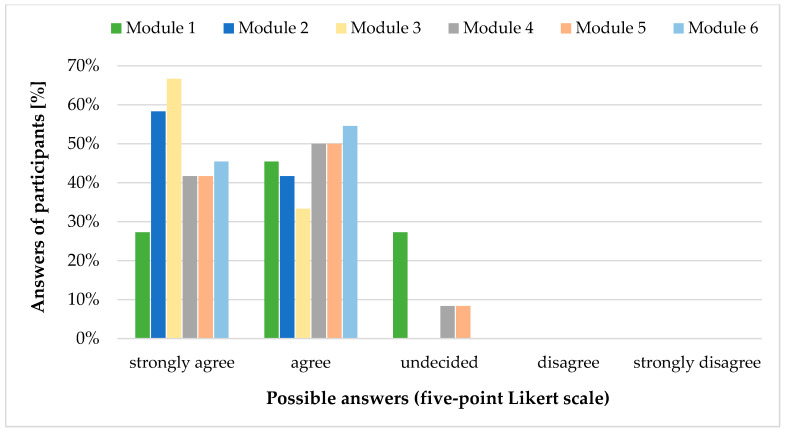
Answers [%] to item GP25: “Please specify for each module whether it contains all the content you would need in everyday clinical practice.” of participants (*n* = 12) of the online user test classified by using a five-point Likert scale. Adapted from [14].

All users declared that Module 2 (Dosing recommendations), Module 3 (Special dosing information), and Module 6 (Formulations) contain all the content they need in every day clinical practice (Figure 1). For the other modules, users had suggestions for improvement, which can be found in Table 3. Except for Module 1 (General information), all modules were rated to be used often to always in clinical practice. Dosing recommendations (Module 2) would be used most frequently. Four participants explicitly mentioned the formulations section when they were asked about the benefits of the database. Reasons were the valuable overview of available formulations (dosage forms, strengths, problematic excipients) in general and information such as flavours of liquid oral formulations in particular.

### 3.3. Second Stage of the Development of the PDIS

The evaluation of existing paediatric formularies revealed that the Dutch Paediatric Formulary (Kinderformularium.nl) was very close to the requirements of the German funding conditions, for example, that the majority of dosing information is based on systematic literature reviews of the primary literature. The Kinderformularium.nl presents as a publicly funded, web-based database containing more than 780 drug monographs. Besides dosing recommendations, it offers additional information, e.g., adverse events and a transparent overview of underlying references. Most importantly, the Kinderformularium.nl already had processes in place in which risk-benefit-analysis based on the best available scientific evidence are performed and reviewed by medical and pharmaceutical experts (editorial board) [12]. The Dutch Kinderformularium also contains an integrated, CE-certified dosing calculator, which was identified as a particularly important functionality during the user test [14].

Considering the evaluation of the prototype presented in this manuscript, the Department of Paediatrics and Adolescent Medicine of the University Hospital of Erlangen and the Foundation of the Dutch Kinderformularium, NKFK (*Nederlands Kenniscentrum Farmacotherapie bij Kinderen*), signed a licensing agreement. Subsequently, a German database “Kinderformularium.de” was established. The content (dosing recommendations, child-specific side effects, contraindications, and warnings) is being translated, revised, and completed according to German requirements, including the results of the present evaluation. The dosing recommendations provided are crosschecked with national and international guidelines aiming at harmonisation across countries. New monographs are being prepared for active ingredients not available in the Netherlands but are used in Germany, e.g., ampicillin, metamizole, and cefixime. The licensing status and child-appropriate pharmaceutical formulations available in the German market are being systematically researched and implemented. Clinical experts from Germany review the drug monographs prior to publication in the PDIS.

The effectiveness of the described PDIS is currently being evaluated as part of an intervention to improve medication safety in a nationwide project (KiDSafe) [15]. As of 2021, the web-based German paediatric formulary will be available free of charge to the German HCP via www.kinderformularium.de.

## 4. Discussion

As global initiatives, particularly PUMA, have failed so far predominantly regarding the availability of generic paediatric medicines, off-label use will remain high for the paediatric population in the future [16]. Therefore, paediatricians need to have access to the best available evidence of the drugs they prescribe.

The evaluation of the prototype of the German PDIS demonstrated that there is a high demand for a national evidence-based paediatric formulary. Dosing recommendations were the most important information for the potential end-users as well as the licensing status and child-appropriate formulations. The prototype of the PDIS appeared adequate to provide the relevant information in terms of content and usability, but deficits still had to be eliminated. This could be achieved via cooperation with an existing formulary in the Netherlands, particularly by using the same technical infrastructure and adding the specific, national requirements in addition to the already established drug information.

### 4.1. Evaluation of the Prototype PDIS

Retrieving evidence-based information on off-label use is time-consuming, as various sources have to be consulted. This procedure is not realistic during daily routine and is one of the reasons for the high variability of dosing regimens in the paediatric population [8,9,10]. Therefore, at first, a prototype of the PDIS was developed and tested early by paediatricians and pharmacists. Despite the limitation of only twelve participants and an average response rate of 50% in the online user test, the results were informative, sufficient, and very helpful for the further development. Moreover, it has previously been demonstrated that with about five subjects, 80% of the usability problems are detected and that additional subjects are less likely to discover new information [17].

At the time of the online user test, the database only contained about 100 drug monographs. This number of monographs was sufficient to illustrate the type of information provided by the PDIS. However, more monographs would have been needed to evaluate the overall benefit for HCP in daily practice. The low amount of available entries was also critically remarked by Plate et al. when they reviewed the risks and benefits of the Zak-Kinderarzneimittel database in their beginnings in 2007 [18,19].

The database already used in clinical practice, which was most often mentioned by all three occupations, was the Rote Liste^®^. The Rote Liste^®^ is a compendium of medicinal products in the German market. The entries are provided by the pharmaceutical companies on a voluntary basis, and all information is based on the official SmPC and package leaflet. Thus, this compendium is on the one hand not complete and on the other hand also not suitable for paediatrics, as it does not address off-label use.

The lack of age-appropriate medicinal products in the market is closely related to high off-label use [20]. Age, health condition, and individual abilities play a significant role in selecting the appropriate dosage form [21]. Nevertheless, prescribers are often unaware of the dosage form, strength, or concentration of the prescribed drugs [22]. This unawareness may lead to the necessity of manipulations to administer the required dose to the patient [23]. Simultaneously, manipulations jeopardise patient and medication safety [24]. Moreover, drugs not licensed for children often contain harmful excipients, especially for young patients [25]. The paediatric formulations section of the PDIS provides users with a comprehensive overview of available dosage forms and strengths, supporting the HCP in choosing the most-appropriate medicinal product. This section was very well perceived by HCP, confirming the importance of formulation aspects in paediatric pharmacotherapy.

A Delphi survey with 31 HCP from more than six continents by Kelly et al. demonstrated that besides dosing information (licensed and off-label indications), formulations, adverse drug reactions, precautions, and drug-drug interactions should be included in a paediatric drug monograph [7]. Particularly the answers to the open-ended questions of the user test helped to adapt the PDIS to the needs of the German end-users, which were in line with the items identified by Kelly et al.

Another important finding of the online user test was the high demand for a dosage calculator. Medication errors constitute a significant health problem in paediatric patients. The risk of adverse drug events following a medication error is higher than in the adult population [26]. Paediatric dosing is very complex, as dosages have to be calculated based on body weight, child’s age or body surface area depending on the drug used. Calculation errors are the most frequent dosing errors with rates up to 28% [27]. Different approaches have been suggested to reduce medication errors, including calculation errors. These are, for example, computerised physician order entry (CPOE) and platforms that contribute to prescribing and administration decisions, including written protocols for high alert drugs [26,28,29]. Recently, van der Zanden et al. showed that a dosage calculator as an add-on to a web-based paediatric formulary has the potential to reduce calculation errors [30]. The integration of an automated dosage calculator, however, implies registration as a medical device, which necessitates the sustainability of the database and liability of the data deployed in the system.

A web-based information platform, like the PDIS, described in this evaluation, provides rapid access to paediatric drug information, aids with dosage calculations and supports decisions on medication processes. The PDIS is applicable at all stages of drug use, providing relevant information not only on dosing but also on the administration, license, and adverse drug profile of the active substance. With its web-based design, it is available for both the out- and inpatient sector, without being dependent on any software of the hospital or practice system and recent (safety) information can be incorporated quickly. Therefore, the PDIS has the potential to improve paediatric drug therapy and to decrease medication errors as long as the provided information is reliable and based on the latest evidence. In addition, the online user test showed that HCP believe that the PDIS has the potential to improve prescribing quality and to decrease adverse drug events. However, sound evidence regarding the impact of the PDIS on medication safety in clinical practice is still lacking [26]. Potential endpoints for this evaluation could be the reduction of medication errors or drug-related hospital admissions. The latter is currently being evaluated in the nationwide KiDSafe study involving twelve German Children’s Hospitals and referring physicians [15].

### 4.2. Further Development of the PDIS

The rigorous specifications for medical devices, both from a technical and a regulatory perspective, were the main reasons why the structure behind the prototype PDIS was found to be inappropriate for further development, and an evaluation of existing paediatric formularies was performed.

One of the best-known paediatric formularies is the British National Formulary for Children (BNFc), which was first published in 2005 and has been regularly updated since [31]. Despite its international reputation, transparency of the references used are missing, and license fees are significantly high.

The SwissPedDose database provides freely accessible dosing recommendations for children in three languages, German, English, and French. Since the project is relatively new, the database is still incomplete. 130 drug monographs are planned to be published by 2022 [32]. The information provided by SwissPedDose is, however, not primarily based on systematic literature searches, but on a consensus-based approach.

The Kinderformularium.nl was considered very close to the purposes of the German formulary, which led to the establishment of a licensing agreement. The cooperation enables the provision of a well-functioning database providing paediatric drug information based on the best available evidence and may be a first step towards a European paediatric formulary. For the first time in the German history of paediatric pharmacotherapy, off-label use is being systematically investigated on a large scale and provided freely accessible to German HCP. The licensing status of the dosing recommendations and the underlying references are stated transparently in order to support HCP as best as possible.

## 5. Conclusions

We have demonstrated that there is a strong demand for a national paediatric formulary with evidence-based dosing recommendations and additional drug information for children in Germany. The evaluation of the prototype PDIS, developed with grants from the Federal Ministry of Health within the action plan to improve the safety of drug therapy in Germany, revealed promising results in terms of usability and content, but also provided limitations. These findings helped us meet the structural and content-related fundamental requirement for a national, publicly accessible PDIS. As of 2021, the web-based paediatric formulary will be available to German HCP via www.kinderformularium.de.

## Figures and Tables

**Table 1:** Modules and information provided by the prototype of the paediatric drug information system (PDIS).

	Modules	Information
1	General information	Pharmacokinetics (child-specific) and pharmacodynamics of the active substance
		Licensing status (also to be found in dosing recommendations), excerpts of the SmPC
		ATC-code, synonyms, brand names
2	Dosing recommendations	Dosing recommendations based on indication, route of administration, and age group
		Licensing status of the dosing recommendations
		References for the dosing recommendations, grade of evidence
3	Special dosing information	Cave-box with vital information Information on dosing adjustment in renal or liver insufficiency
4	Interactions	Clinically relevant interactions
5	Side effects and warnings	Child-specific adverse drug events, contraindications, warnings and precautions Recommended action in case of overdose
6	Formulations	Overview of the available dosage forms and formulations in the German market
		Examples of medicinal products suitable for use in children
		Selected formulation-based information, e.g., excipients, flavours, divisibility

SmPC: Summary of Product Characteristics.

**Table 2:** Descriptive analysis of participants and summarised free-text answers classified by professional group.

Professional Group	Physicians in Private Practice	Clinicians	Pharmacists
Participants (n (%))	6 (50)	3 (25)	3 (25)
Response rate (%)	43	50	75
Professional experience Mean (years)	22	7	6
Prescriptions ^1^/ Directions for use ^2^ per day Mean (n)	27	11	15
Computer knowledge/skills ^3^ Mean (Median)	2.2 (2)	2.7 (3)	3 (3)
How satisfied are you with the currently available information on pharmacotherapy for children? ^4^	3.5	3.0	3.3
Databases already used ((n) if > 1)	Rote Liste^®^ (2) [Red list], fachinfo.de^®^ [service for SmPC], dosage handbooks, e.g., DGPI Handbuch [handbook of the German Society for Paediatric Infectiology], Drug information systems of the practice-software (e.g., Ifap^®^, duria^®^, albis^®^), guidelines	Rote Liste^®^ [Red list], fachinfo.de^®^ [service for SmPC], dosage handbooks, e.g., Medikamente in der Pädiatrie (Elsevir) [medicines in paediatrics], Neofax^®^ (micromedex), Pädiatrische Dosistabellen (Thieme Verlag) [paediatric dosing tables], Arzneimittel kompakt (Thieme Verlag) [drugs compact], Tabellarium nephrologicum (M. Daschner)	Rote Liste^®^ [Red list], Fachinformation [SmPC], Embryotox^®^ (2) [Pharmacovigilance- and consultation centre for embryonic toxicology], ABDA-Datenbank^®^ (2) [German drug information system by AVOXA^®^], Dosing.de^®^ [Information platform for dosing in renal failure]

^1^ Physician in private practice/clinician; ^2^ pharmacist; ^3^ 1 = low, 2 = middle, 3 = high; ^4^ 1 = very satisfied, 2 = rather satisfied, 3 = partly, 4 = rather dissatisfied, 5 = very dissatisfied.

**Table 3:** Answers from open-ended questions of the online user test clustered by categories.

Category	N (%)	Details (Examples)
Search function	7 (23)	Include indications, international brand names, or dosage forms in the search function
Cross-links	5 (17)	Include cross-links to alternative drugs (e.g., antibiotics for otitis media) or formulations
Layout	7 (23)	Highlighting of essential facts, e.g., side effects, warnings; uniform presentation of dosing tables
Content		
Module 1	1 (3)	Licensing status missing in one specific monograph
Module 2	2 (7)	Include duration of treatment, weight-based dosing lists, dosage calculator
Module 4	1 (3)	Include background information for interactions
Module 5	2 (7)	Include child-specific adverse event
Module 6	2 (7)	Include package size
Others	3 (10)	Include additional monographs and date of the last amendment of the monograph

N: frequency; %: percentage.

## Data Availability

The data presented in this study are available on request from the corresponding author.
